# Dynamic 3D Point-Cloud-Driven Autonomous Hierarchical Path Planning for Quadruped Robots

**DOI:** 10.3390/biomimetics9050259

**Published:** 2024-04-24

**Authors:** Qi Zhang, Ruiya Li, Jubiao Sun, Li Wei, Jun Huang, Yuegang Tan

**Affiliations:** 1 School of Mechanical and Electronic Engineering, Wuhan University of Technology, Wuhan 430070, China; zq3521584689@whut.edu.cn (Q.Z.); jubiaosun@163.com (J.S.); weili@whut.edu.cn (L.W.); ygtan@whut.edu.cn (Y.T.); 2 Robotics and Intelligent Manufacturing Engineering Research Center of Hubei Province, Wuhan 430070, China; 3 School of Information Engineering, Wuhan University of Technology, Wuhan 430070, China

**Keywords:** quadruped robots, 3D point cloud, complex terrain, dynamic obstacles, particle swarm optimization (PSO), artificial potential field (APF), dynamic window approach (DWA)

## Abstract

Aiming at effectively generating safe and reliable motion paths for quadruped robots, a hierarchical path planning approach driven by dynamic 3D point clouds is proposed in this article. The developed path planning model is essentially constituted of two layers: a global path planning layer, and a local path planning layer. At the global path planning layer, a new method is proposed for calculating the terrain potential field based on point cloud height segmentation. Variable step size is employed to improve the path smoothness. At the local path planning layer, a real-time prediction method for potential collision areas and a strategy for temporary target point selection are developed. Quadruped robot experiments were carried out in an outdoor complex environment. The experimental results verified that, for global path planning, the smoothness of the path is improved and the complexity of the passing ground is reduced. The effective step size is increased by a maximum of 13.4 times, and the number of iterations is decreased by up to 1/6, compared with the traditional fixed step size planning algorithm. For local path planning, the path length is shortened by 20%, and more efficient dynamic obstacle avoidance and more stable velocity planning are achieved by using the improved dynamic window approach (DWA).

## 1. Introduction

In recent years, quadruped robots, due to their excellent motion flexibility and terrain adaptability, have been extensively developed to play important roles in many fields, such as military, disaster relief, factory inspection, etc. [1,2]. Path planning is a crucial component of the quadruped robot for accomplishing the above tasks. It is usually separated into two steps: (1) global path planning using a known environment map, and (2) local path planning using real-time perception of the local environment. Currently, numerous algorithms have been proposed to plan safe and reliable paths for wheeled robots and quadrotor unmanned aerial vehicles (UAVs) [3,4,5,6]. However, the inherent limitations of these algorithms include (1) the absence of performance validation in a real environment point cloud map, (2) scale difficulties in path smoothness calculation, and (3) the lack of evaluation of the velocity of dynamic obstacles and future trajectory for dynamic obstacle avoidance. Furthermore, for the quadruped robot platform, the limitations of these algorithms are apparent in the lack of terrain complexity evaluation, which results in a risk of unstable robot motion. Therefore, a hierarchical path planning approach for quadruped robots with a particle swarm optimization (PSO)-based 3D artificial potential field (APF) and improved dynamic window approach (DWA) is proposed in this article to break through the inherent limitations of existing path planning methods for quadruped robots and boost the autonomous adaptation of quadruped robots to complex environments.

### 1.1. Global Path Planning

The existing global path planning algorithms can be grouped into two classes: heuristic algorithms (rapidly exploring random tree, D*, A*, APF, etc.) and intelligence algorithms (artificial neural network, genetic algorithm, particle swarm optimization, reinforcement learning, etc.). A search tree with the starting point as the root node is constructed by the rapidly exploring random tree-based algorithms [7,8,9], and the feasible path is found by the single-query algorithm. However, these algorithms are computationally expensive and struggle to construct optimal smooth paths in a point cloud map. The D*-based and A*-based algorithms [4,10,11,12] incorporate the heuristic function into the Dijkstra algorithm [5] to plan optimal paths in 2D space. However, these algorithms suffer from the “dimensional disaster” in 3D space, resulting in extremely inefficient planning. The artificial neural network-based algorithms [13,14,15,16,17] explicitly render the configuration space and the robot state-space into an array of locally connected neurons. This array is then trained with various methods, resulting in a path that connects the present state of the robot with the target state. However, these algorithms suffer from a challenging training process. The genetic algorithms [18,19] and PSO-based algorithms [20,21] represent alternative solutions of the optimization function as individuals of a population, and they evolve the population according to the fitness value of the individuals to select a more suitable population. However, the terrain complexity is not considered in the optimization objective function of these algorithms, which makes them inapplicable to quadruped robots. The reinforcement learning-based algorithms [22,23,24,25] utilize environmental spatiotemporal information and set a reward structure to maximize the value function to plan optimal paths. However, when the point cloud map is used as the input, the reward becomes sparse, which increases the training times of these algorithms and decreases their planning efficiency.

The APF-based algorithms [26,27] simulate repulsive and attractive fields to plan the direction of robot motion, as a general and easy-to-implement framework for global path planning. However, traditional APF algorithms suffer from fixed repulsive and attractive force coefficients, which cause robots to fail to reach the target point and stop when there are obstacles near the target point, and even to oscillate when the robots fall into the minimum trap of the local potential field. For upgrading the traditional APF, the PSO-based APF algorithms [28,29] are proposed to optimize the repulsive and attractive force coefficients. The fitness function of PSO includes two indicators: distance to the target, and path smoothness. However, the efficiency of global planning is severely limited by the fixed step size used in these methods. Moreover, the absence of a path complexity indicator makes these algorithms limited in applications to quadruped robots. In [30], obstacles are approximated as regular geometric shapes and projected onto a 2D plane. The fitness function is calculated as the linear sum of the iteration number of particles and the difference in steering angle of the neighboring path points. However, as the number of optimization iterations rises, the significant of the difference in magnitude between different optimization indicators will lead to a tendency to update the particle’s position and velocity during the optimization process. In [31], opposition-based learning (OBL) is introduced to improve the inertia weight and step size to prevent the precocity of PSO. However, a problem arises with slow convergence or even non-convergence, resulting in low planning efficiency. In [32], the tangent vector, which is based on the information about obstacles, is added to the APF model as an auxiliary force in the obstacle avoidance process. However, the tangent vector is challenging to estimate in real complex environments.

### 1.2. Local Path Planning

The existing local path planning algorithms mainly include behavior decomposition (BD), cased learning (CL), and the dynamic window approach (DWA). The BD-based algorithms [33] decompose the path planning into independent units, i.e., behavioral primitives, which collaborate to accomplish the entire movement task. However, due to the limited weight and space of quadruped robots, it is difficult for them to carry numerous sensors and actuators. An intelligent typical case-based reasoning for path planning is proposed in the CL-based algorithms [34,35]. The path is planned based on current empirical knowledge and road network information. The reliability of such algorithms is difficult to assess, as they rely on empirical knowledge.

The DWA-based approaches [36,37] have attracted the attention of a wide range of researchers. The DWA strategy aims to select the optimal combination of velocities in the dynamic velocity window by minimizing the evaluation function. In [38,39], the evaluation functions of these approaches are calculated based only on the static environmental information, making them unsuitable for planning in dynamic environments. An improved DWA approach for quadruped robots is proposed in [40], where the emergency obstacle avoidance goal and the nearest obstacle are distinguished to achieve segmentation design of collision probability coefficients for static and dynamic obstacles. However, dynamic obstacles are only evaluated in terms of their positional impact, while the potential risk of collision caused by the influence of dynamic obstacles in terms of impact velocity is ignored. In [41], arc-shaped obstacles are gridded, and concave obstacles are made convex in order to prevent the robot from becoming trapped in the obstacle. The evaluation function is adaptively updated according to the robot’s safety threshold, obstacles, and environment information. The influence of the dynamic obstacles’ state on the efficiency and stability of planning is ignored in these algorithms. In addition, convex processing of the environment is not applicable in complex real-world environments, and the tiny grid size reduces the efficiency of local path planning.

In summary, traditional path planning algorithms for quadruped robots face the following issues:

(1) The environment map, composed of idealized regular geometry, is used as the input of the algorithm, which cannot effectively plan the path in real complex environments.
(2) The planning efficiency is limited due to the fixed step size used by PSO-based APF algorithms. The terrain complexity is ignored in the evaluation function of the fitness function, which poses a risk of planning an unreliable path. The calculation of the terrain potential field is ignored in the 3D APF algorithms, resulting in the inability of the quadruped robot’s torso to maintain an appropriate height from the ground.
(3) The influence of the velocity of dynamic obstacles is ignored in the local path planning algorithm, which decreases the efficiency and stability of the local planning.
(4) The optimal velocity planning based on DWA algorithms is limited in solving velocity, due to the vast size of the point cloud.

To solve the above problems, a hierarchical path planning method consisting of PSO-based 3D APF and improved DWA is proposed in this article. The PSO-based 3D APF algorithm is utilized for global path planning, while a point cloud height segmentation-based calculation method for the terrain potential field and a DEM-based terrain complexity calculation method are proposed. An improved DWA algorithm is employed for local path planning. The velocity of dynamic obstacles is mapped to their distance from the robot, which is used to predict the potential collision area. Then, a strategy for temporary target point selection is proposed. Finally, CUDA is used to accelerate the solution velocity in path planning.

The main contributions of our approach are as follows:

(1) The neighborhood points of the quadruped robot’s torso are segmented into obstacle points and terrain points. Using a static environment point cloud map to plan the global path, the spatial shape features and data distribution features are preserved well, which helps the robot to choose the optimal path.
(2) The terrain potential field is introduced into the APF to restrict the distance between the torso and the ground to ensure that the torso remains within a stable operating altitude range, thereby guaranteeing the reliability of path planning.
(3) The terrain complexity is integrated into the fitness function to enhance the reliability of global path planning. The method of calculating path smoothness is improved to overcome the scale problem.
(4) A method of predicting the potential collision area is proposed to enhance the efficiency and stability during dynamic obstacle avoidance. The calculation of the optimal velocity combination is accelerated by CUDA.

The outline of this article is as follows: Section 2 presents the methodological framework of the proposed hierarchical path planning method. Section 3 illustrates the map pre-processing: (1) point cloud processing, and (2) point cloud height segmentation. The approach proposed for global and local path planning is discussed in Section 4 and Section 5, respectively. In Section 6, the experimental details are illustrated and the results are analyzed. Section 7 concludes the article and highlights future research directions.

## 2. Methodological Framework

**Figure 1:**
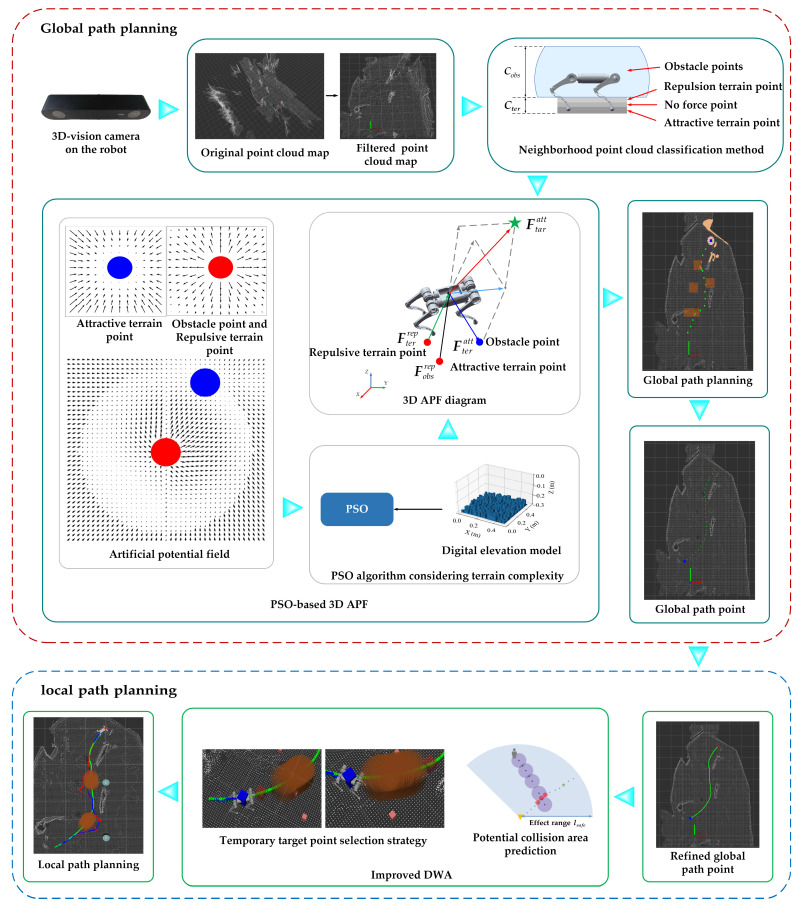
The developed hierarchical path planning method.

The overall framework of the developed hierarchical path planning is illustrated in Figure 1. Global path planning and local path planning are conducted separately. An offline planning mode is adopted in the global path planning, and a static environment point cloud map is utilized as the input. The point cloud is segmented into obstacle points and terrain points based on height and distance to the robot. The terrain points are utilized to calculate the terrain potential field. Thus, the total potential field is the sum of the obstacles, the target point, and the terrain potential field. The global path points will be planned in the direction of the fastest decreasing potential field gradient with the 3D APF algorithm. In this case, the force parameters of the potential field and the step size will be optimized by the PSO algorithm.

An online planning mode with a real-time environment point cloud is utilized as the input in the local path planning. The global path points are refined and selected as temporary target points for local path planning based on the strategy for temporary target point selection. A pedestrian tracking algorithm is utilized to predict the potential collision area. The velocity of dynamic obstacles is mapped to their distance from the robot in the evaluation function of the improved DWA algorithm. The optimal velocity combination, whose solution is accelerated by CUDA, is planned by the improved DWA algorithm.

## 3. MAP Pre-Processing

### 3.1. Environment Point Cloud Processing

The environment point cloud map consists of the static environment point cloud map generated by the algorithm [42] and the real-time local environment perception [43,44]. The static environment map is used in global path planning. The following sequence is applied to crop the raw environment map and reduce noise:

- A voxel filter with leaf size $l_{l}$ is applied to reduce the size of points;
- A statistical outlier removal (SOR) filter with a neighborhood radius of $l_{r}$ and a neighborhood point number of $l_{n}$ is utilized to reduce the number of outliers;
- A passthrough filter is used to crop the raw environment map along specified dimensions.

The real-time local environment perception is used in local path planning. The above processing sequence is also applied to real-time local environment information first, and then the scope of the point cloud of the local environment is cropped to reduce unnecessary calculations in local path planning by the following sequence:

- The point cloud at the depth limit $l_{d}$, representing the influence range of obstacles, is cropped during local path planning;
- The point cloud at the height $l_{f}$ is cropped to remove the ceiling points;
- The algorithm in [43] is used to track the motion state of dynamic obstacles.

The motion state of dynamic obstacles is denoted by $X_{\mathrm{dyobsmot}}^{c}=\left[X_{\mathrm{dyobs}}^{c},V_{\mathrm{dyobs}}^{c}\right]$, where $X_{\mathrm{dyobs}}^{c}\in R^{3}$ and $V_{\mathrm{dyobs}}^{c}\in R^{3}$ represent the position and velocity of the dynamic obstacle c($c=0,\ldots ,N_{\mathrm{dyobs}}$), respectively.

### 3.2. Height Segmentation of the Point Cloud

**Figure 2:**
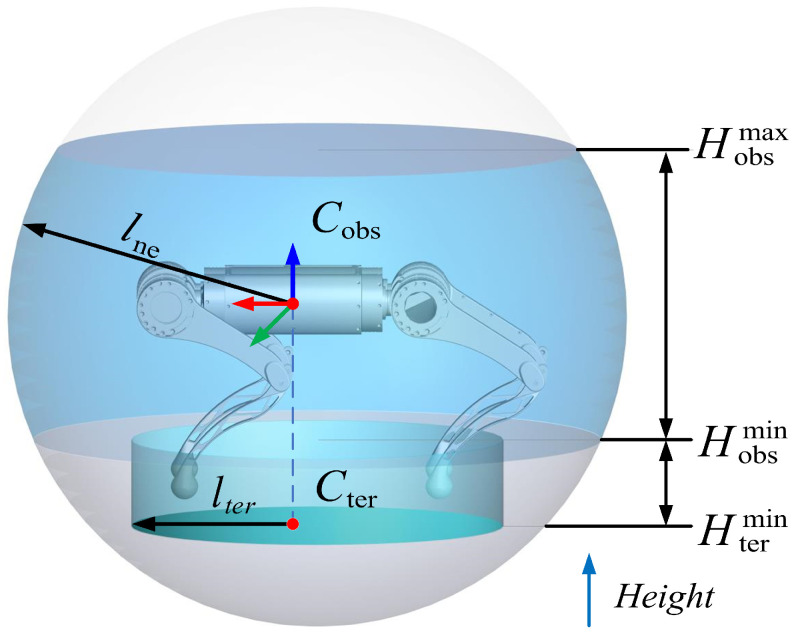
Neighborhood point cloud classification.

The purpose of the point cloud segmentation is to obtain the terrain and obstacle points in the neighborhood point cloud. The schematic segmentation diagram is shown in Figure 2. The neighborhood points of the robot are defined as points in a sphere with a radius of $l_{\mathrm{ne}}$. The sphere is concentric with the center of mass of the robot’s torso. The obstacle points and terrain points are denoted by $C_{\mathrm{obs}}$ and $C_{\mathrm{ter}}$, respectively. The obstacle points $C_{\mathrm{obs}}$ are defined as points within height from $H_{\mathrm{obs}}^{\min}$ to $H_{\mathrm{obs}}^{\max}$ in the sphere. The terrain points $C_{\mathrm{ter}}$ are defined as points within a cylinder with a radius of $l_{\mathrm{ter}}$ and a height from $H_{\mathrm{ter}}^{\min}$ to $H_{\mathrm{obs}}^{\min}$. Attractive and repulsive forces are exerted on the robot by the terrain points, depending on the height of the point, to keep the robot’s body at a proper height. The detailed calculation is described in Section 4.

## 4. Global Path Planning with PSO-Based 3D APF

**Figure 3:**
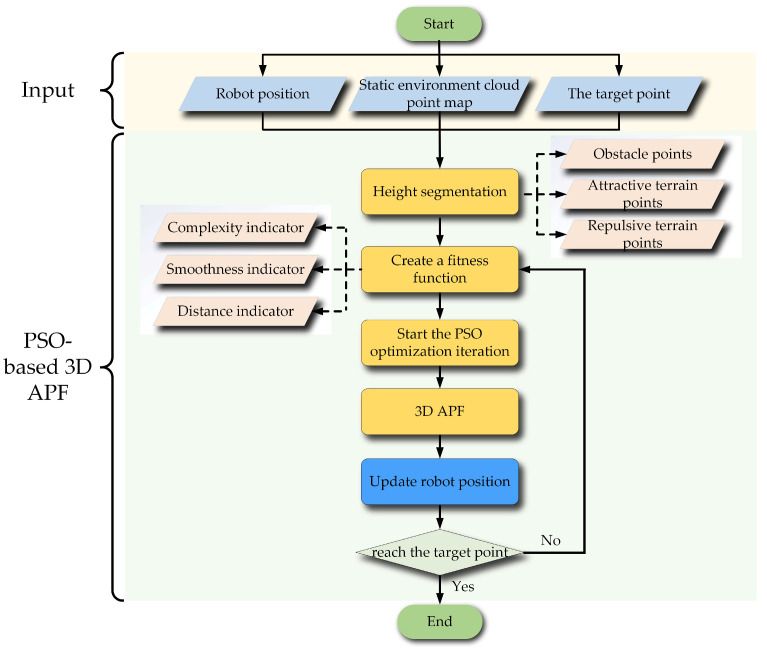
The designed PSO-based 3D APF.

In this section, firstly, the basic forms of attractive and repulsive potential field calculation for the 3D APF are illustrated, and the corresponding potential fields are calculated. Then, the parameters to be optimized in the 3D APF are illustrated. Finally, the fitness function, each indicator, and the proposed terrain complexity and improved path smoothness calculation method are introduced. The framework of the designed PSO-based 3D APF is shown in Figure 3.

### 4.1. Three-Dimensional (3D) APF with Terrain Potential Field

Referring to the traditional APF algorithm, attractive and repulsive potential field functions of the 3D APF can be expressed in the following form:

(1) $$U_{\mathrm{att}}(X)=\frac{K_{\mathrm{att}}}{2}d^{2}(X_{\mathrm{att}},X)$$

(2) $$U_{\mathrm{rep}\;}\left(X\right)=\left\{\begin{array}{cc} \frac{K_{\mathrm{rep}\;}}{2}{\left(\frac{1}{d\left(X,X_{\mathrm{rep}\;}\right)}-\frac{1}{\rho_{\mathrm{rep}\;}}\right)}^{2}d^{n}\left(X_{\mathrm{att}\;},X\right) & d\left(X,X_{\mathrm{rep}\;}\right)⩽\rho_{\mathrm{rep}\;}\\ 0_{3\times 1} & d\left(X,X_{\mathrm{rep}\;}\right)>\rho_{\mathrm{rep}\;} \end{array}\right.$$

where $X\in R^{3}$ is the position of the center of mass of the quadruped robot’s torso. $X_{\mathrm{att}}\in R^{3}$ and $X_{\mathrm{rep}}\in R^{3}$ are the positions of the objects exerting attractive and repulsive forces, respectively. $K_{\mathrm{att}}={\left[K_{\mathrm{att}}^{x},K_{\mathrm{att}}^{y},K_{\mathrm{att}}^{z}\right]}^{T}$ and $K_{\mathrm{rep}}={\left[K_{\mathrm{rep}}^{x},K_{\mathrm{rep}}^{y},K_{\mathrm{rep}}^{z}\right]}^{T}$ are the distance gain coefficient of the attractive and repulsive potential field in the x−y−z directions, respectively. $U_{\mathrm{att}}\left(X\right)={\left[U_{\mathrm{att}}^{x}\left(X\right),U_{\mathrm{att}}^{y}\left(X\right),U_{\mathrm{att}}^{z}\left(X\right)\right]}^{T}$ is defined as a three-dimensional vector. $U_{\mathrm{att}}^{x}(X)$, $U_{\mathrm{att}}^{y}(X)$, and $U_{\mathrm{att}}^{z}(X)$ represent the attractive potential under the distance gain coefficient in each direction. $U_{\mathrm{rep}}(X)$ is similar to $U_{\mathrm{att}}\left(X\right)$. The advantage of our method is that the attractive and repulsive forces in each direction can be controlled separately through three distance gain coefficients. $d\left(X_{\mathrm{att}},X\right)$ represents the Euclidean distance between the center of mass of the quadruped robot’s torso and the object exerting an attractive force. $\rho_{rep}$ is defined as the maximum distance at which the robot is affected by obstacles, while n is the index factor of the repulsive potential field.

According to Equation (1), the attractive potential field of the target point can be expressed as follows:

(3) $$U_{\mathrm{att}}^{\mathrm{tar}}\left(X\right)=\frac{K_{\mathrm{att}}^{\mathrm{tar}}}{2}d^{2}\left(X_{\mathrm{att}}^{\mathrm{tar}},X\right)$$

where $K_{\mathrm{att}}^{\mathrm{tar}}\in R^{3}$ is the distance gain coefficient of the attractive potential field of the target point, while $X_{\mathrm{att}}^{\mathrm{tar}}\in R^{3}$ is the position of the target point.

According to Equation (2), the repulsive potential field of the obstacle point cloud can be expressed as follows:

(4) $$U_{\mathrm{rep}}^{P_{i}}\left(X\right)=\frac{K_{\mathrm{rep}}^{\mathrm{obs}}}{2}{\left(\frac{1}{d\left(X,X_{P_{i}}^{\;\;}\right)}-\frac{1}{\rho_{\mathrm{obs}}}\right)}^{2}d^{n}\left(X_{\mathrm{att}}^{\mathrm{tar}},X\right)$$

where $K_{\mathrm{rep}}^{\mathrm{obs}}\in R^{3}$ is the distance gain coefficient of the repulsive potential field of the obstacle point. $X_{P_{i}}^{\;\;}\in R^{3}$ is the position of the obstacle point. $P_{i}$ represents the obstacle points in the neighborhood, and $\rho_{\mathrm{obs}}$ is defined as the maximum distance at which the robot is affected by obstacles.

During the iterative planning process, the quadruped robot’s torso is subject to the repulsive force exerted by each obstacle point in the neighborhood. This article superimposes the repulsive potential field of each obstacle point and takes the average value as the repulsive potential field of obstacle points in the neighborhood. Therefore, the repulsive potential field of the obstacle points can be expressed as follows:

(5) $$U_{\mathrm{rep}}^{\mathrm{obs}}\left(X\right)=\left[\sum_{i=1}^{N_{\mathrm{obs}}}U_{\mathrm{rep}}^{P_{i}}\left(X\right)\right]/N_{\mathrm{obs}}$$

where $N_{\mathrm{obs}}$ is the number of obstacle points in the neighborhood.

During the motion of the quadruped robot, the height of the torso’s center of mass from the ground should be kept within an appropriate range. The minimum and maximum height thresholds are represented by $H_{\mathrm{com}}^{\min}$ and $H_{\mathrm{com}}^{\max}$, respectively. $H_{\mathrm{com}}$ is the height of the center of mass of the robot’s torso. When $H_{\mathrm{com}}$> $H_{\mathrm{com}}^{\max}$, the attractive terrain point will attract the robot. Similarly, when $H_{\mathrm{com}}$< $H_{\mathrm{com}}^{\min}$, the repulsive terrain point will repulse the robot. According to the point cloud height segmentation, the terrain point cloud exerts two forces on the robot’s torso at the same time. The attractive force is generated by the attractive point $C_{\mathrm{att}}$, while the repulsive force is generated by the repulsive force point $C_{\mathrm{rep}}$. The attractive potential field $U_{\mathrm{att}}^{q_{j}}\left(X\right)$ and the repulsive potential field $U_{\mathrm{rep}}^{w_{k}}\left(X\right)$ can be expressed as follows:

(6) $$\left\{\begin{array}{cc} U_{\mathrm{att}}^{q_{i}}\left(X\right)=\frac{K_{\mathrm{att}}^{\mathrm{ter}}}{2}\left|H_{\mathrm{com}}-H_{q_{j}}\right|\cdot V\left(X_{q_{i}},X\right) & H_{\mathrm{com}}>H_{\mathrm{com}}^{\max}\\ U_{\mathrm{rep}}^{w_{k}}\left(X\right)=K_{\mathrm{rep}}^{\mathrm{ter}}\left|\frac{2}{\left|H_{\mathrm{com}}-H_{w_{k}}\right|}-\frac{1}{\left(H_{\mathrm{com}}^{\min}-H_{\mathrm{com}}^{\max}\right)}\right|\cdot \\ \left|H_{\mathrm{com}}-H_{w_{k}}\right|\cdot V\left(X,X_{w_{k}}\right) & H_{\mathrm{com}}<H_{\mathrm{com}}^{\min} \end{array}\right.$$

Similar to Equation (5), we superimpose the attractive potential field and the repulsive potential field generated by the terrain points, taking the average value as the attractive potential field and the repulsive potential field generated by the ground:

(7) $$\left\{\begin{array}{l} U_{\mathrm{att}}^{\mathrm{ter}}\left(X\right)=\left[\sum_{j=1}^{N_{\mathrm{att}}^{\mathrm{ter}}}U_{\mathrm{att}}^{q_{j}}\left(X\right)\right]/N_{\mathrm{att}}^{\mathrm{ter}}\\ U_{\mathrm{rep}}^{\mathrm{ter}}\left(X\right)=\left[\sum_{k=1}^{N_{\mathrm{rep}}^{\mathrm{ter}}}U_{\mathrm{rep}}^{w_{k}}\left(X\right)\right]/N_{\mathrm{rep}}^{\mathrm{ter}} \end{array}\right.$$

where $N_{att}^{ter}$ and $N_{\mathrm{rep}}^{\mathrm{ter}}$ are the size of attractive terrain points and repulsive terrain points, respectively. $q_{j}$ is the jth, $j=1,\ldots ,N_{\mathrm{att}}^{\mathrm{ter}}$, point in attractive terrain points, and $w_{k}$ is the kth, $k=1,\ldots ,N_{\mathrm{rep}}^{\mathrm{ter}}$, point in repulsive terrain points. $H_{q_{j}}$ and $H_{w_{k}}$ are the heights of the jth attractive terrain point and the kth repulsive terrain point, respectively; their positions are denoted by $X_{q_{j}}\in R^{3}$ and $X_{w_{k}}\in R^{3}$, respectively. $V\left(\cdot \right)\in R^{3}$ is the unit direction vector of the center of mass of the robot’s torso and the terrain point. The direction is from the terrain point to the center of mass of the robot’s torso when the terrain point is repulsive; otherwise, the direction of $V\left(\cdot \right)$ is the opposite. $K_{\mathrm{att}}^{\mathrm{ter}}\in R^{3\times 3}$ is a diagonal matrix. The elements on the diagonal are the distance gain coefficients of the potential field of attractive terrain points in the x−y−z directions. $K_{\mathrm{rep}}^{\mathrm{ter}}\in R^{3\times 3}$ is similar to $K_{\mathrm{att}}^{\mathrm{ter}}\in R^{3\times 3}$. Therefore, the total potential field generated by the static environment point cloud can be expressed as follows:

(8) $$U=U_{\mathrm{att}}^{\mathrm{tar}}\left(X\right)+U_{\mathrm{rep}}^{\mathrm{obs}}\left(X\right)+U_{\mathrm{att}}^{\mathrm{ter}}\left(X\right)+U_{\mathrm{rep}}^{\mathrm{ter}}\left(X\right)$$

According to the classical APF principle, the robot moves in the direction of downward potential energy. The direction of movement can be represented by a unit vector, as follows:

(9) $$F=-\nabla \left(U\right)$$

The location update of global path planning can be expressed as follows:

(10) $$X_{t+1}=X_{t}+F\cdot step$$

where $X_{t}$ and $X_{t+1}$ are the position of the center of mass of the quadruped robot’s torso at the current iteration time and the next iteration time, respectively. *Step* is the planning step size, that is, the Euclidean distance between $X_{t}$ and $X_{t+1}$.

### 4.2. PSO-Based Optimization of APF

During global path planning, the potential field parameters and the steps planned need to be updated dynamically. The PSO algorithm is utilized to optimize these parameters adaptively. PSO is a population-based stochastic optimization algorithm whose particles are represented as potentially optimal solutions in a D-dimensional search space. The ith particle’s position is defined as $z_{i}={\left[K_{\mathrm{att}}^{\mathrm{tar}},K_{\mathrm{att}}^{\mathrm{ter}},K_{\mathrm{rep}}^{\mathrm{ter}},K_{\mathrm{rep}}^{\mathrm{obs}},step,n\right]}^{T}\in R^{14\times 1}$ and its velocity is denoted by $v_{i}\in R^{14\times 1}$. The position and velocity of the particles are updated as follows:

(11) $$v_{i}=c_{0}v_{i}+c_{1}r_{1}\left(z_{\mathrm{pdi}}-z_{i}\right)+c_{2}r_{2}\left(z_{\mathrm{cgi}}-z_{i}\right)\;z_{i}=z_{i}+v_{i}$$

where $c_{0}$ is the inertial weight; $c_{1}$ and $c_{2}$ are the individual and group learning factors that satisfy the condition $c_{1},c_{2}\in \left[0,2\right]$, respectively; $r_{1},r_{2}\in \left[0,1\right]$ are random factors; $z_{\mathrm{pdi}}$ is the position with the best fitness for the ith particle so far; and $z_{\mathrm{cgi}}$ is the position with the best fitness for all particles in the current iteration.

### 4.3. Fitness Function

To overcome the limitations of the classical APF, the PSO algorithm is utilized to optimize the attractive and repulsive parameters. In order to improve the effectiveness of this method in quadruped robots, a new fitness function is proposed in this article, which includes a terrain complexity evaluation.

The fitness function is composed of three evaluation indicators: the distance from the robot to the target point, path smoothness, and terrain complexity, which can be expressed as follows:

(12) $$F_{\cos t}^{m}=\alpha_{1}\cdot F_{1}^{m}\left(X_{t},X_{\mathrm{tar}}\right)+\alpha_{2}\cdot F_{2}^{m}\left(X_{t-1},X_{t},X_{t+1}\right)+\alpha_{3}\cdot F_{3}^{m}\left(X_{t},step_{m}\right)$$

where $F_{\cos t}^{m}$ represents the fitness function of the mth particle. n is the number of iterations. $F_{1}^{m}\left(X_{t},X_{\mathrm{tar}}\right)$, $F_{2}^{m}\left(X_{t-1},X_{t},X_{t+1}\right)$, and $F_{3}^{m}\left(X_{t},step_{m}\right)$ are the distance indicator, path smoothness indicator, and terrain complexity indicator, respectively. $X_{t-1}$, $X_{t}$, and $X_{t+1}$ are the position of the robot in the t−1th, tth, and t+1th iteration, respectively. $\alpha_{1}$, $\alpha_{2}$, and $\alpha_{3}$ are the weight coefficients of each indicator, respectively. $step_{m}$ is the planning step in the m th particle’s position information.

The distance indicator is calculated from the Euclidean distance between the robot’s torso and the target point, which can be expressed as follows:

(13) $$F_{1}^{m}=||X_{t}-X_{\mathrm{tar}}|{|}_{2}$$

To improve the efficiency of global path planning, a dynamic step size is designed and implemented in our method. In traditional methods, path smoothness is represented as the angle between neighboring steps, easily resulting in scaling problems when using dynamic programming steps. To solve this problem, we use the ratio of the valid move length to the actual move length as the path smoothness. The improved path smoothness indicator can be expressed as follows:

(14) $$F_{2}^{m}=\frac{||X_{t}-X_{t-1}|{|}_{2}+||X_{t+1}-X_{t}|{|}_{2}-||X_{t+1}-X_{t-1}|{|}_{2}}{||X_{t+1}-X_{t-1}|{|}_{2}}$$

**Figure 4:**
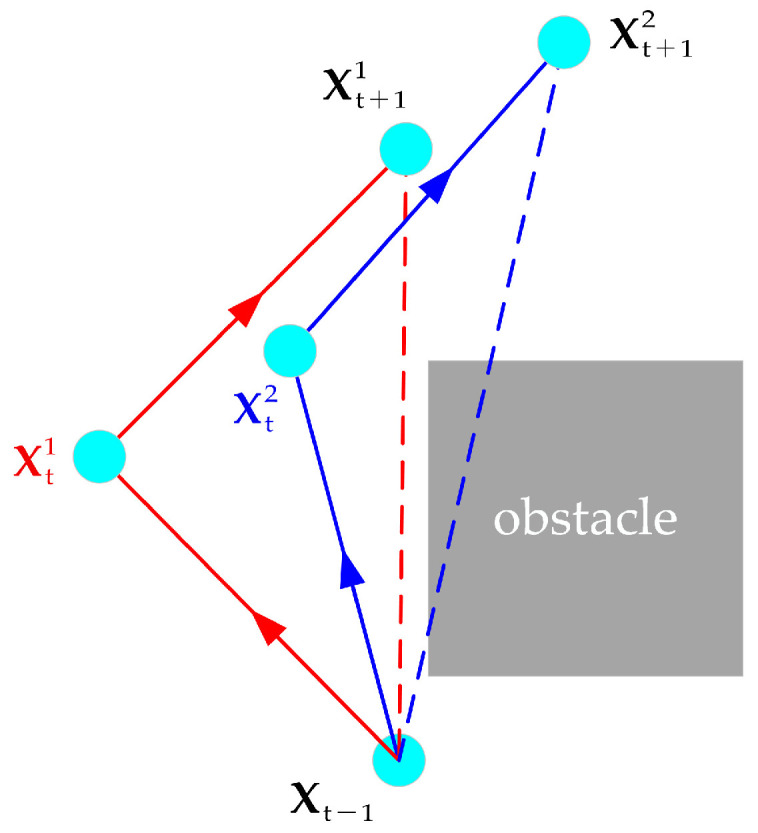
The position of the robot in the t−1th, tth, and t+1th iterations under different planning paths.

The valid move length is defined as the sum of the Euclidean distance between $X_{t-1}$ and $X_{t}$ and the Euclidean distance between $X_{t}$ and $X_{t+1}$ minus the Euclidean distance between $X_{t-1}$ and $X_{t+1}$. As shown in Figure 4, we can substitute $X_{t-1}$, $X_{t}^{1}$, $X_{t+1}^{1}$ and $X_{t-1}$, $X_{t}^{2}$, $X_{t+1}^{2}$ into Equation (13) to compare the path smoothness of the red path and the blue path, respectively. The smaller the calculation result of Equation (14), the smoother the path will be. When three path points are on a straight line, Equation (14) obtains the minimum value 0, and the path is the smoothest.

**Figure 5:**
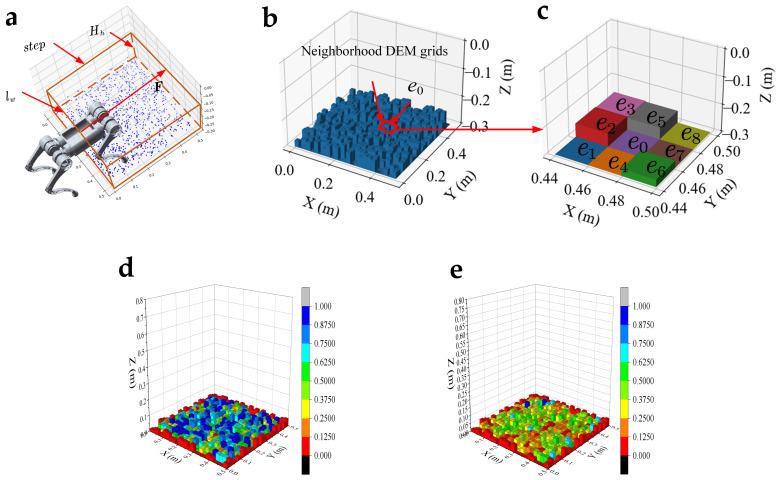
The terrain complexity calculation: (**a**) the rectangular region (within the yellow area); (**b**) the regular grid of the digital elevation model; (**c**) the 3×3 DEM grid; (**d**,**e**) the calculation results of roughness and undulation, respectively, on the simulated point cloud.

The terrain complexity is determined by its roughness and undulation. For calculating terrain complexity, the center of mass of the robot’s torso is taken as the starting point, and the point cloud is cropped in a rectangular region with a vertical length of step, a horizontal length of $l_{w}$, and a height of $H_{h}$, in the F direction. The rectangular region is shown in Figure 5a. In the rectangular area, the height difference between the point cloud (blue) and the robot’s torso is distributed in $\left[-0.3,-0.25\right]$ m. The raw point cloud is represented by a digital elevation model (DEM) composed of regular grids. The DEM grids are shown in Figure 5b. The terrain complexity is expressed as the average of the roughness and undulation of the DEM grids. As shown in Figure 5c, a DEM grid denoted by *e*_0_ and its neighboring DEM grids denoted by *e*_1_~*e*_8_ are selected. For convenience, the height of each DEM grid is also denoted by $e_{i}$. The roughness is defined as the sum of the absolute value of the height difference between the grid *e*_0_ and its neighboring grids. The roughness can be calculated by Equation (15):

(15) $$R=\sum_{j=1}^{N_{DEM}}\left[\frac{1}{8}\sum_{i=1}^{8}\mathrm{abs}\left(e_{i}^{j}-e_{i}^{0}\right)\right]$$

where $N_{DEM}$ is the size of THE DEM in the rectangular region, and $j=1,\ldots ,N_{DEM}$ is the index of DEMs. The undulation is defined as the sum of the absolute value of maximum height difference between the grid $e_{0}$ and its neighboring grids. The undulation can be calculated by Equation (16):

(16) $$A\;=\sum_{j=1}^{N_{DEM}}\underset{i=1,\ldots ,8}{\max}\left(\left|e_{i}^{j}-e_{0}^{j}\right|\right)$$

Therefore, the terrain complexity can be calculated by Equation (17):

(17) $$F_{3}^{m}=\beta_{1}\cdot R+\beta_{2}\cdot A$$

where $\beta_{1}$ and $\beta_{2}$ are the weighting coefficients of the roughness and undulation, respectively.

## 5. Local Path Planning with Improved DWA

**Figure 6:**
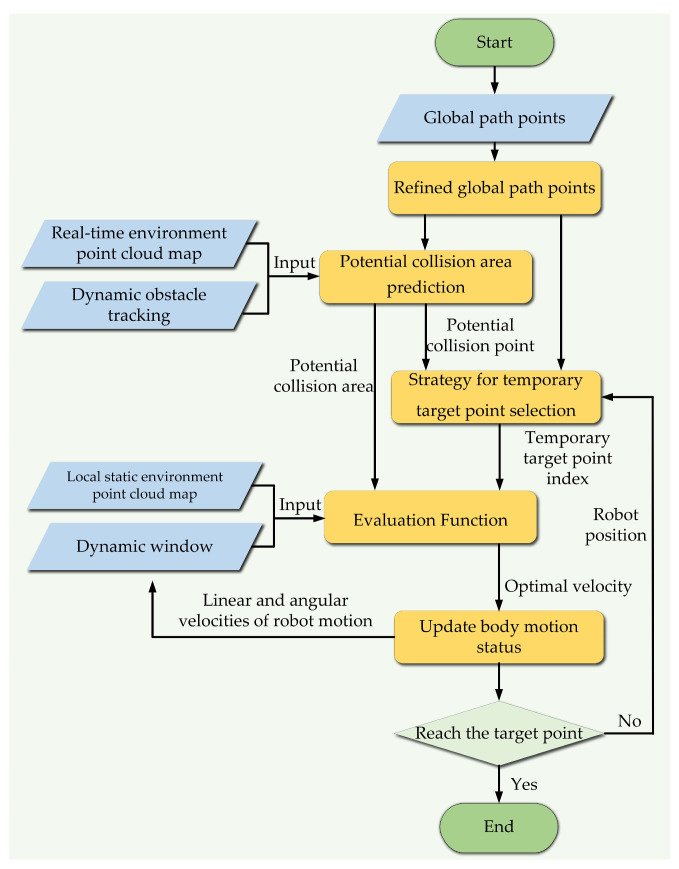
The improved DWA.

In this section, the method for predicting potential collision areas is first illustrated. Then, the strategy for selecting temporary target points is introduced. Finally, the related evaluation function is constructed, which takes the effect of the velocity of dynamic obstacles into consideration. The overall framework of the improved DWA is shown in Figure 6.

### 5.1. Potential Collision Area Prediction

**Figure 7:**
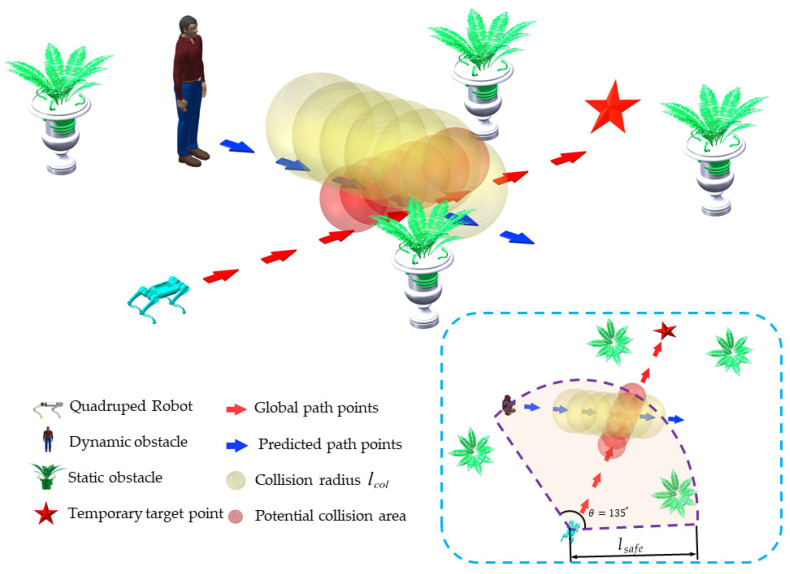
Prediction of potential collision area (red area).

The schematic diagram of potential collision area prediction is shown in Figure 7. Firstly, the safe range $l_{\mathrm{safe}}$ is utilized to determine whether a dynamic obstacle affects local path planning. When the distance between the robot and a dynamic obstacle is less than the safe range, the collection of future path points of the dynamic obstacle denoted by $C_{\mathrm{dyobs}}$ in a prediction period $T_{p}$ will be predicted according to the motion state of the dynamic obstacle. The future path points are predicted dynamically in real time. This means that the path points will be predicted at each moment based on the current position of the dynamic obstacle, assuming that it moves at a constant velocity.

Therefore, track point prediction can be expressed as follows:

(18) $$\begin{array}{c} X_{\mathrm{dyobs}}^{i}=X_{\mathrm{dyobs}}^{0}+i\cdot V_{\mathrm{dyobs}}^{0}\;\;\;i=0,\ldots ,\left[\frac{T_{p}}{N_{\mathrm{dyobs}}}\right] \end{array}$$

where $X_{\mathrm{dyobs}}^{0}\in R^{3}$ and $V_{\mathrm{dyobs}}^{0}\in R^{3}$ are the position and velocity of the dynamic obstacle in the world coordinate system at the current moment, respectively; the symbol $\left[\cdot \right]$ represents rounding; $N_{\mathrm{dyobs}}$ is the number of future path points. The collection of future path points of dynamic obstacle $C_{\mathrm{dyobs}}$ can be expressed as follows:

(19) $$\begin{array}{cc} C_{\mathrm{dyobs}}=\left\{X_{\mathrm{dyobs}}^{i},\ldots \right\} & i=0,\ldots ,\left[\frac{T_{p}}{N_{\mathrm{dyobs}}}\right] \end{array}$$

where $X_{\mathrm{dyobs}}^{i}$ is the future path point of the dynamic obstacle.

The risk area is generated with the future path point of the dynamic obstacle as the center, which means that when the robot is within the risk area, there will be a risk of collision with the dynamic obstacle. The radius of the risk area is $l_{\mathrm{col}}$. When the global path point planned in the global path planning experiment is located in the risk area, it is considered to be a potential collision point. $C_{\mathrm{col}}$ is the index of all potential collision points. A virtual obstacle with a radius $r_{\mathrm{virobs}}$ is added at each potential collision point. The function of virtual obstacles is to help the robot avoid potential collision areas as much as possible, but they cannot completely prevent the robot from entering.

### 5.2. Strategy for Temporary Target Point Selection

An important concept of hierarchical path planning is that the local path planning must be guided by global path points. Thus, in local path planning, the temporary target points are points selected from the global path points to prevent the robot from colliding with dynamic obstacles.

Firstly, the global path points outputted by the PSO-based 3D APF algorithm need to be refined to reduce the distance between path points, which is beneficial to achieve more reliable local planning. The refinement of global path points is achieved through linear interpolation between neighboring path points, which can be expressed as follows:

(20) $$\begin{array}{cc} X_{i}=\frac{X_{t+1}-X_{t}}{N_{p}}\cdot i+X_{t} & i=1,\ldots ,N_{p} \end{array}$$

where $X_{t}\in R^{3}$ and $X_{t+1}\in R^{3}$ are the positions of neighboring global path points. $X_{i}\in R^{3}$ represents path points obtained by linear interpolation. $N_{p}$ represents the numbers of interpolation points.

Due to the existence of dynamic obstacles, there are situations where temporary target points can be potential collision points. Therefore, it is necessary to design a strategy for temporary target point selection to guide the robot to avoid dynamic obstacles safely.

**Figure 8:**
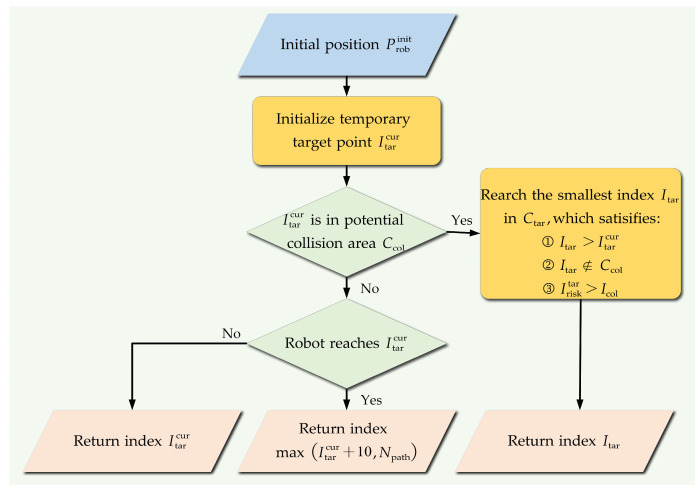
The selection strategy of temporary target points.

The temporary target points are selected as shown in Figure 8. $I_{\mathrm{tar}}$ is the index of the temporary target point in global path points. $C_{\mathrm{tar}}$ is the collection of refined global path points.

The process of the strategy for temporary target point selection is as follows:

1. The initial position of the robot in the world coordinate system is recorded as $P_{\mathrm{rob}}^{\mathrm{init}}$. The path point closest to $P_{\mathrm{rob}}^{\mathrm{init}}$ among the global path points is selected as the initial temporary target point, and its index in the global path points is recorded as $I_{\mathrm{tar}}^{\mathrm{cur}}$.
2. The potential collision area prediction method proposed in Section 5.1 is used to determine whether the current temporary target point is a potential collision point.
3. If the current temporary target point is not a potential collision point, it is necessary to further determine whether the robot reaches the temporary target point. If the robot does not reach the current temporary target point, the index of the current temporary target point will be returned; otherwise, the returned index can be expressed as follows:
  (21) $$I_{\mathrm{tar}}^{\mathrm{cur}}=\max\left(I_{\mathrm{tar}}^{\mathrm{cur}}+10,N_{\mathrm{path}}\right)$$
  where $N_{\mathrm{path}}$ is the number of refined global path points.

If the temporary target point is a potential collision point, the point is abandoned and a global path point in the collection $C_{\mathrm{tar}}$ is reselected as the temporary target point. The reselected temporary target point is recorded as $I_{\mathrm{tar}}^{\;\;}$, which should meet the following conditions:

(1) The index of the reselected temporary target point $I_{\mathrm{tar}}^{\;\;}$ is greater than the index of the abandoned temporary target point $I_{\mathrm{tar}}^{\mathrm{cur}}$;
(2) The minimum distance from the reselected temporary target point to all potential collision areas is $l_{\mathrm{risk}}^{\mathrm{tar}}$, which should be greater than the radius of the risk area $l_{\mathrm{col}}$;
(3) The reselected temporary target point is not a potential collision point;
(4) The reselected temporary target point is the global path point in the collection $C_{\mathrm{tar}}$ with the smallest index that satisfies the above conditions.

### 5.3. Evaluation Function

Based on the robot’s current motion state and each set of speed combinations, the future path points of the torso at time $T_{b}$ are predicted. The set of future path points is recorded as $C_{b}$. The average distance between the path points and the temporary target point is calculated as the distance evaluation indicator; the calculation method of this index can be expressed as follows:

(22) $$\begin{array}{c} Dist_{\mathrm{tar}}=\frac{1}{N_{C_{b}}}\cdot \sum_{i=1}^{N_{C_{b}}}||X_{C_{b}^{i}}-X_{\mathrm{tar}}|{|}_{2}\;\;\;i=1,\ldots ,N_{C_{b}} \end{array}$$

where $N_{C_{b}}$ is the number of path points of the torso at time $T_{b}$, $X_{C_{b}^{i}}$ is the position of the i th path point, and $X_{\mathrm{tar}}$ is the position of the temporary target point with index $I_{\mathrm{tar}}^{\;\;}$.

According to the potential collision area prediction method in Section 5.1, it can be seen that there are three types of obstacles in the environment, namely, static, dynamic, and virtual obstacles. The DWA algorithm is improved by mapping the velocity of dynamic obstacles to its distance from the robot. The evaluation function can be expressed as follows:

(23) $$l_{\mathrm{dyobs}}^{i}=\exp\left(\frac{V_{\mathrm{dyobs}}\cdot \left(X_{C_{b}^{i}}-X_{\mathrm{dyobs}}\right)}{||X_{C_{b}^{i}}-X_{\mathrm{dyobs}}|{|}_{2}}\right)\cdot ||X_{C_{b}^{i}}-X_{\mathrm{dyobs}}|{|}_{2}\;\;\;i=1,\ldots ,N_{C_{b}}$$

where $X_{\mathrm{dyobs}}$ is the position of the dynamic obstacle in the world coordinate system. $V_{\mathrm{dyobs}}$ represents the velocity of the dynamic obstacle, which is the adjustment factor of the actual distance between the robot and the dynamic obstacle. When the velocity of the dynamic obstacle is greater, the distance score between the dynamic obstacle and the robot increases. By minimizing the evaluation function, the robot can safely avoid dynamic obstacles. The velocity of dynamic obstacles is included in the evaluation function, which is beneficial to improving the stability of speed planning. The minimum distance between the robot and dynamic obstacles can be expressed as follows:

(24) $$\begin{array}{cc} {\mathrm{Dist}}_{\mathrm{dyobs}}=\min\left(l_{\mathrm{dyobs}}^{i}\right) & i=1,\ldots ,N_{C_{b}} \end{array}$$

The impact of virtual obstacles on the evaluation calculation is similar to that of static obstacles. The different is that the position of virtual obstacles changes with the motion state of the dynamic obstacle. Virtual obstacles will not exclude the robot from entering the potential collision area, but when the robot is close to the potential collision area they will increase the evaluation score of the distance between the robot and the static obstacle. The distance between the robot and the static obstacle can be expressed as follows:

(25) $$\begin{array}{c} {\mathrm{Dist}}_{\mathrm{obs}}=\left\{\begin{array}{cc} \begin{array}{c} ln\left(e-\min\left(||X-X_{\mathrm{col}}^{m}|{|}_{2}\right)-r_{\mathrm{virobs}}\right)\cdot \\ \min\left(||X_{C_{b}^{i}}-X_{P_{j}}|{|}_{2}\right) \end{array} & N_{\mathrm{col}}\geq 1\\ \min\left(||X_{C_{b}^{i}}-X_{P_{j}}|{|}_{2}\right) & N_{\mathrm{col}}=0 \end{array}\right.\\ i=1,\ldots ,N_{C_{b}},j=1,\ldots ,N_{\mathrm{obs}},m=1,\ldots ,N_{\mathrm{col}}\; \end{array}$$

where $X_{\mathrm{col}}^{m}$ is the position of the mth potential collision point, and $N_{\mathrm{col}}$ is the number of potential collision points.

The DWA evaluation function is used to select the speed combination with the smallest score in the dynamic window, that is, the optimal speed combination. The optimal speed selected through the evaluation function is used to update the motion status of the fuselage, including position, speed, and orientation. The score of the DWA evaluation function can be expressed as follows:

(26) $$G\left(v,\omega \right)=w_{1}\cdot {\mathrm{Dist}}_{\mathrm{tar}}\left(v,w\right)+w_{2}\cdot {\mathrm{Dist}}_{\mathrm{dyobs}}\left(v,w\right)+w_{3}\cdot {\mathrm{Dist}}_{\mathrm{obs}}\left(v,w\right)$$

where $w_{1}$, $w_{2}$, and $w_{3}$ are the weighting factor of each evaluation indicator. v and ω are the linear and angular velocity, respectively, which are selected from the dynamic velocity window $V_{r}$ as expressed in Section 6.

## 6. Experimental Results and Discussion

### 6.1. Experimental Platform and Setup

**Figure 9:**
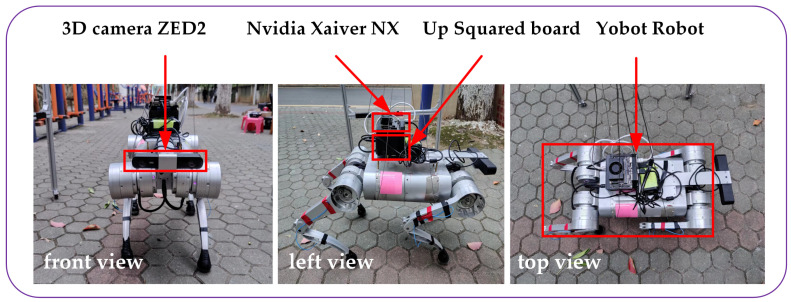
Experimental setup.

A commercial quadruped robot (Y10, manufactured by YOBTICS from Shandong Province, China) was utilized as the experimental platform, as shown in Figure 9. For environment reconstruction and dynamic obstacle tracking, a 3D camera (ZED2, manufactured by Stereolabs from San Francisco, CA, USA) with an IMU sensor was mounted on the front of the robot. For real-time performance, a single-board computer (UP Squared Board, manufactured by AAEON from Jiangsu Province, China) and an embedded system-on-module (Nvidia Xavier NX, manufactured by Nvidia from Santa Clara, CA, USA) were mounted on the back of the robot’s torso. The Nvidia Xavier NX was utilized to reconstruct the static environment, generate real-time point clouds, and track dynamic obstacles. The UP Squared Board was utilized to plan both global and local paths. The communication method between the Nvidia Xavier NX and the UP Squared Board depends on ROS (Robot Operating System). The desired control commands generated by local path planning are transformed to the robot control board via LCM (Lightweight Communications and Marshalling).

**Figure 10:**
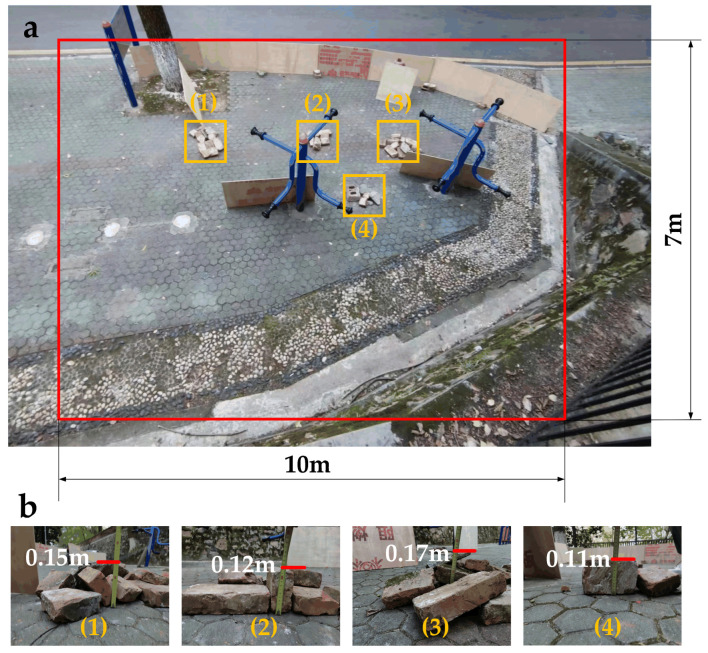
Static environment setup: (**a**) the rectangle-shaped environmental area with 4 brick piles; (**b**) the height of each brick pile.

The experimental environment is shown in Figure 10. The shape of the experimental environment was equivalent to a rectangle with a length of 10 m and a width of 7 m. The rectangle-shaped experimental area is shown in Figure 10a. Bricks were used to augment the complexity of the terrain. There were four brick piles stacked on the terrain, as shown in Figure 10b. The maximum height of the brick piles was between 0.11 m and 0.17 m.

**Figure 11:**
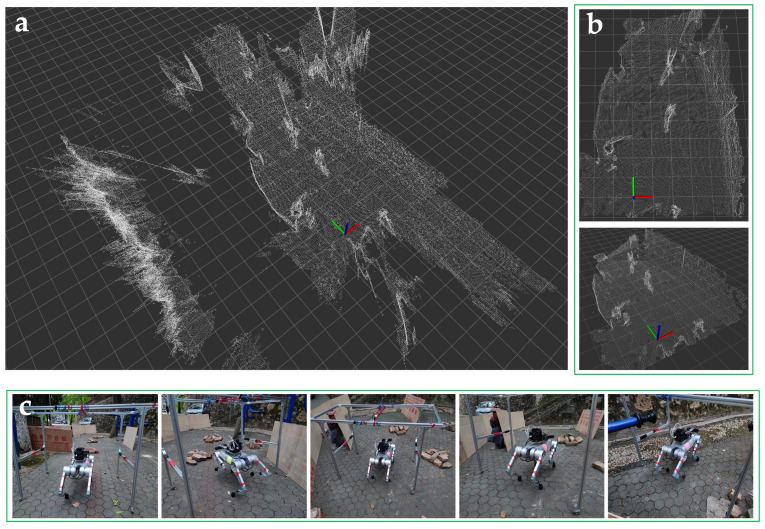
Static environment point cloud map: (**a**) the raw point cloud map; (**b**) the filtered map; (**c**) the process of constructing the map.

This article utilizes the point cloud generated by the 3D camera to create a static environment point cloud map. The process of constructing the static environment point cloud map is shown in Figure 11. The raw static environment point cloud map with 259,162 points is shown in Figure 11a. The origin of the world coordinate system was selected as the initial camera position when constructing the map.

As discussed in Section 3, the map needs to be filtered to reduce the noise and the number of point clouds. The pre-processing flow is as follows:

- The passthrough filter is applied to crop the map in artificially set x−y−z directions and ranges; only the point cloud within the scope of the test site is kept. The passthrough filter parameters are set as follows: $l_{x}\in \left[-2,5\right]\;m$, $l_{y}\in \left[-1,10\right]\;m$, $l_{z}\in \left[-0.3,2.5\right]\;m$.
- A voxel filter with leaf size $l_{l}=0.4\;m$ is utilized to reduce the size of points;
- A statistical outlier removal filter with the number $l_{n}=20$ is utilized to reduce outliers of neighborhood points within a radius $l_{r}=0.4\;m$.

The number of point clouds contained in the pre-processed map reduced from 259,162 to 33,945, as shown in Figure 11b.

### 6.2. Results and Discussion for PSO-Based 3D APF in Global Path Planning

The range of parameters to be optimized for the PSO-based 3D APF is shown in Table 1. The fixed parameters of the PSO algorithm and global path planning are shown in Table 2. The population size of PSO was 100. According to the characteristics of the quadruped robot’s motion, the vertical distance from the torso to terrain points was set to $H_{com}^{res}=\left[0.25,0.35\right]$ m.

The initial position of the robot in the world coordinate system was set to $\left[-0.5,1.5,0.1\right]$. Two target destinations in the world coordinate system were set in our experiment: $T_{1}=\left[0,7.5,0\right]$ and $T_{2}=\left[2,8,0\right]$. In the experiment for evaluating terrain complexity, the weight factors of the roughness and undulation were set as *β*_1_ = *β*_2_ = 1. Four sets of weight parameters $\alpha =\left[\alpha_{1},\alpha_{2},\alpha_{3}\right]$ of the fitness function were applied to each target point, which were set to [1, 0, 0], [1, 5, 0], [1, 0, 50], and [1, 5, 50], respectively.

**Figure 12:**
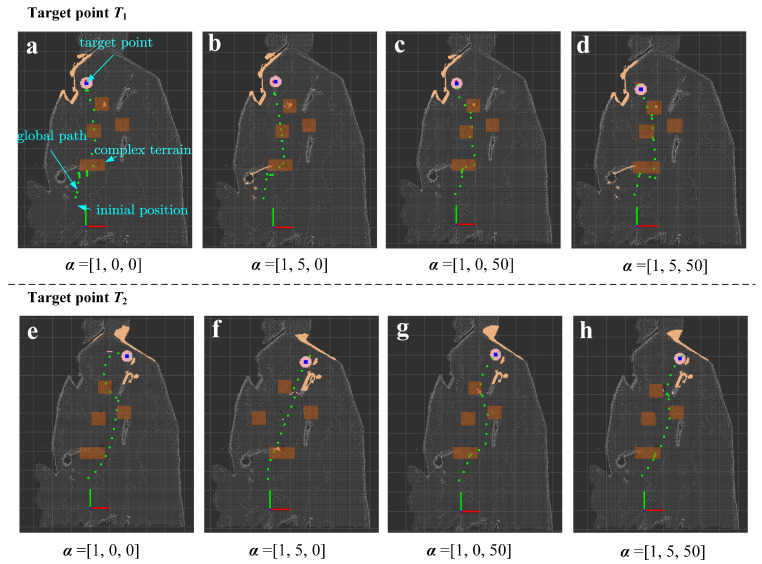
The results of global path planning with target points $T_{1}$ and $T_{2}$.

The path planning process was visualized by utilizing ROS and RViz (Robot Visualization). The results of global path planning with the target points $T_{1}$ and $T_{2}$ are shown in Figure 12.

The average and maximum values of path smoothness and terrain complexity were utilized as metrics to compare the performance of global path planning with different parameter settings. The quantitative comparison of the PSO-based 3D APF’s performance under different parameter settings is shown in Table 3. It is obvious that when the parameter is set to $\alpha_{2}=5$, the average and maximum values of the smoothness of the global path are the smallest. Likewise, when the parameter is set to $\alpha_{3}=50$, the average and maximum values of the terrain complexity are the smallest. Undeniably, setting the parameters simultaneously to $\alpha_{2}=5$ and $\alpha_{3}=50$ only achieves the best performance in path smoothness with the target $T_{1}$; otherwise, the performance is suboptimal, albeit still close to the best performance. The biggest difference between the suboptimal and best performance is within 5%. It is believed that the reason for this is that the designed fitness function needs to adaptively balance each indicator. By comparing the performance of different parameter settings, the path smoothness can be improved by the proposed fitness function to guide the robot to pass over the low-complexity terrain.

To compare the results (planning efficiency and path smoothness) of our method with those of the method in reference [29], the weight parameters of the fitness function were set to $\alpha =\left[\alpha_{1},\alpha_{2},\;\alpha_{3}\right]=\left[1,5,50\right]$. In reference [29], a fixed step was used for PSO. The efficiency of planning is reflected in the number of PSO iterations, with fewer iterations indicating higher efficiency. The effective step ratio is used as an overall gauge for the path smoothness. The effective step ratio is calculated as follows:

(27) $$\sigma =\frac{L/step_{f}}{iter-L/step_{f}}$$

where iter is the number of iterations, L is the linear distance between the initial position and the target point, and $step_{f}$ is the fixed step.

**Figure 13:**
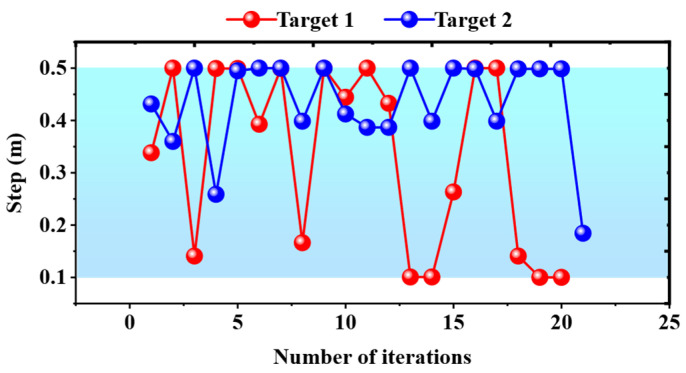
Variation in step after being optimized by PSO.

Obviously, a larger effective step ratio means that the global path is closer to a straight line, which also represents a smoother global path. The step variation is shown in Figure 13. The step was adaptively adjusted between 0.1 and 0.5 m. The effective step ratio was calculated by using the average of steps in all iterations. The fixed step was set to five sets of values from 0.1 to 0.5 m. The effective step ratio is shown in Table 4. As can be seen from the table, our method obtains a higher effective step ratio, with a maximum improvement of 9/0.67 ≈ 13.4 times and an average improvement of (9/0.67 + 3.2/0.43)/2 ≈ 10.4 times. Furthermore, our method requires fewer iterations, resulting in a maximum improvement of 121/21 ≈ 5.8 times and an average improvement of (121/21 + 103/20)/2 ≈ 5.5 times. The experimental results illustrate that our method can improve both path planning efficiency and path smoothness.

**Figure 14:**
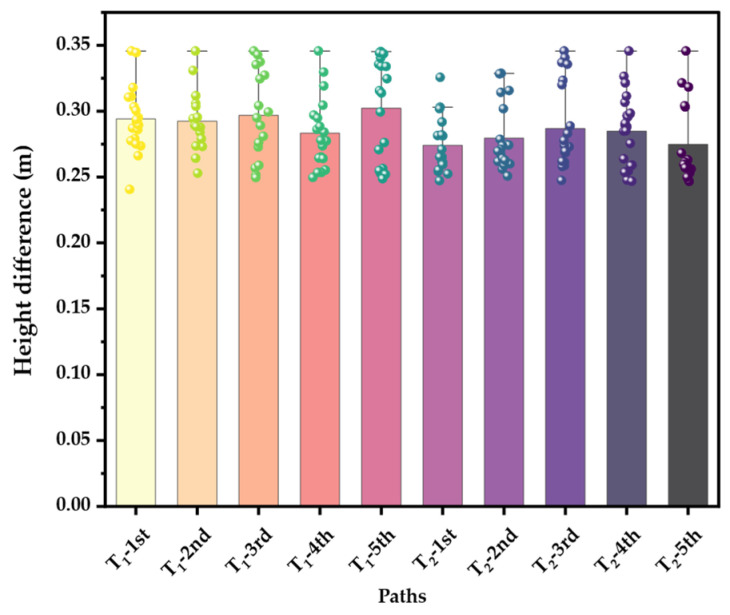
The height difference between the path points and the ground.

The height of global path points above the ground was used as the metric to evaluate the path reliability. The ground height is represented by the average height of the terrain point cloud in the neighborhood. The global path planning was repeated five times to check the height difference. It can be seen from Figure 14 that the height of the robot’s torso from the ground was maintained within the range $H_{com}^{res}\in \left[0.24,0.35\right]$ m, comparable to the maximum height of the attractive point and the minimum height of the repulsive point. Thus, the validity and rationality of the method for constraining the height of the torso using the terrain point potential field were verified.

### 6.3. Results and Discussion for Improved DWA in Local Path Planning

Firstly, on the basis of traditional DWA algorithms, this article proposes a method of potential collision area prediction and a strategy for temporary target point selection. In the evaluation function of the improved DWA, the velocity of the dynamic obstacle is mapped to its distance from the robot to improve the efficiency and stability of the robot’s dynamic obstacle avoidance. Then, our improved DWA is compared with the traditional DWA algorithm described in reference [40]. Since our improved DWA algorithm focuses on dynamic obstacle avoidance, we emphatically compare the efficiency of dynamic obstacle avoidance and the stability of velocity planning between the two algorithms. Finally, the computational scale of the optimal velocity solution is briefly introduced, and the solution velocity is accelerated by CUDA.

The velocity window is essentially a space of achievable velocities that are determined by the robot’s current motion state and motion parameters within a given time interval. According to the kinematic and dynamic constraints of our quadruped robot, the motion parameters of the quadruped robot are listed in Table 5. In the following experiment, the time interval was set to 0.1 s.

**Figure 15:**
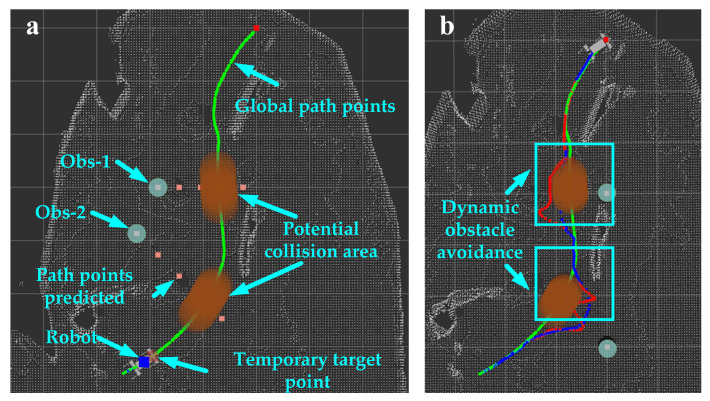
Dynamic obstacle avoidance: (**a**) potential collision area prediction; (**b**) the local path planning results of the improved DWA and traditional DWA algorithms. The green paths represent the refined global path points. The red and blue paths represent the planning results of the improved DWA and traditional DWA algorithms, respectively. The paths in the cyan box are the dynamic obstacle avoidance process.

In the local velocity planning experiment, the dynamic obstacle tracking was applied to detect the position and velocity of dynamic obstacles. Potential collision areas were predicted, and temporary target points were selected by setting two dynamic obstacles, obs-1 and obs-2. The effective range of the dynamic obstacles was set to $l_{\mathrm{safe}}=3$ m. At each tracking output time, assuming that dynamic obstacles move in a straight line at a constant velocity, the path points at the future time $T_{p}=3$ s were predicted based on the position and velocity of the dynamic obstacles. With the path points of the dynamic obstacles as the center, a risk area with a radius $l_{\mathrm{col}}=0.35$ m was generated. The virtual obstacles were added, with a radius $r_{\mathrm{virobs}}=0.35$ m. The predicted results of the potential collision region are shown in Figure 15a. The potential collision area was applied to the temporary target point selection and evaluation function. The local path planning results of the improved DWA and traditional DWA algorithms are shown in Figure 15b. The performance evaluation of the Z-axis linear velocity was ignored. The efficiency of dynamic obstacle avoidance is expressed as the length of the locally planned path. The stability of the velocity planning was determined by calculating the average of the velocity variance across all iteration windows, each with a size set to 5.

**Figure 16:**
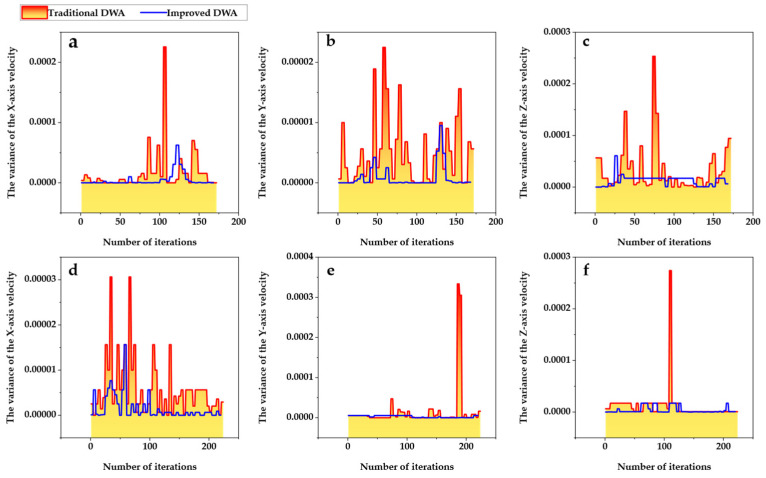
The velocity planning results of the improved DWA and traditional DWA algorithms. Only the linear velocity of the body in the X and Y directions, along with the angular velocity around the Z-axis, is compared. (**a**–**c**) Obstacle 1. (**d**–**f**) Obstacle 2.

The velocity planning results of the improved DWA method and the traditional DWA method are shown in Figure 16. Based on the results of velocity planning, the stability and efficiency of the two methods were compared in this study. The stability of dynamic obstacle avoidance can be assessed by calculating the average variance in velocity within a given iteration window. The stability of velocity planning increases as the mean value decreases. The size of the iteration window was set to 5, meaning that the variance within the iteration window was calculated for every five adjacent combinations of iteration velocity. The efficiency of dynamic obstacle avoidance is represented by the length of the local path planning. The results of the local path planning are shown in Table 6. The stability of the torso’s X- and Y-axis velocities, along with the planned angular velocities around the Z-axis, is compared in this table. It can be observed from the table that the variance of the improved DWA algorithm in each velocity component and the combined velocity is smaller, with a minimum improvement ratio of 2.73 times. This suggests that the improved algorithm can effectively improve the velocity stability during dynamic obstacle avoidance. Similarly, the planned path length during dynamic obstacle avoidance is effectively reduced, with a reduction ratio of more than 20%.

**Figure 17:**
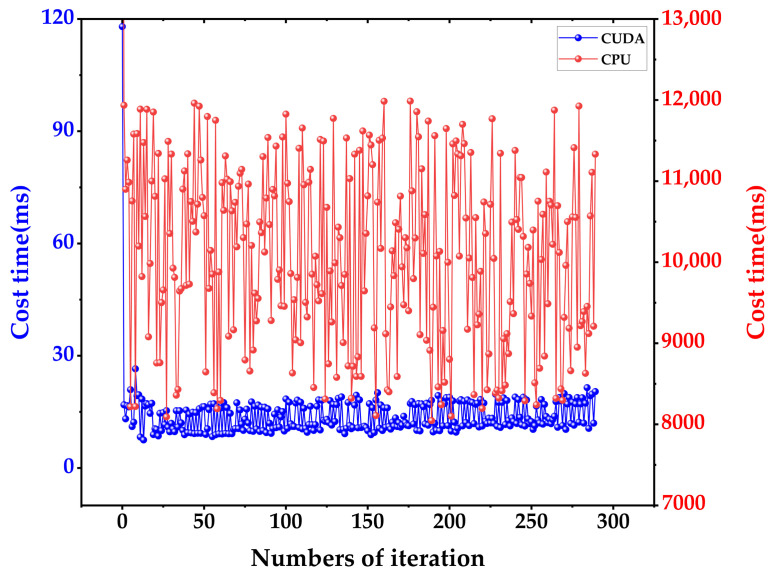
The cost time of evaluation function calculation with CUDA and CPU.

Taking the 3D point cloud of the actual environment as the input of the path planning algorithm is the biggest difference between our work and most other path planning works. When the 3D point cloud is combined with velocity information, millions of calculations are generated. This makes it difficult for the algorithm to achieve good real-time performance when calculating the expected velocity required for the robot to move at the next moment. To this point, CUDA was employed instead of a traditional CPU to accelerate the calculation velocity in our study. One velocity combination within the velocity window corresponds to one CUDA thread running independently. Information on the point cloud, velocity combinations, robot’s current position, target point position, and dynamic obstacles is utilized as input for each thread calculation. In each thread, a score is calculated for each velocity combination, and an array of scores is returned with the evaluation function. Finally, the score array is sorted to obtain the index of velocity combinations corresponding to the highest score. In actual deployment, the block size of the CUDA kernel function is set to 128, and the grid size is set to 6. The memory size occupied by the kernel function during operation is about 300 MB. The calculation time of the evaluation function before and after CUDA acceleration was compared by the experiment. The cost time of evaluation function calculation with CUDA and CPU is shown in Figure 17.

## 7. Conclusions

Path planning is an essential procedure for robots to move autonomously in complicated dynamic environments. For quadruped robots, most of the proposed global path planning methods lack terrain complexity assessment, while the local path planning methods do not fully consider practical factors like dynamic obstacles’ moving velocity. In this study, a 3D point-cloud-driven hierarchical path planning method was developed for quadruped robots, which consists of a PSO-based 3D APF for global path planning and improved DWA for local path planning. The main contributions of our results are as follows:

(1) In global path planning, the authors improved the calculation method of path smoothness to make it suitable for variable step optimization. Compared with the traditional APF method using a fixed step size, the dynamic step planning method that we propose is more effective in terms of the number of iterations and the step rate to achieve the optimal performance, effectively enhancing the planning efficiency.
(2) In global path planning, a terrain complexity calculation method based on a digital elevation model is proposed, and the terrain complexity evaluation is designed in the PSO fitness function. Compared with the PSO evaluation function that does not evaluate terrain characteristics, the developed algorithm is more efficient in complex environments. It is more advantageous for robots to plan movements on complex terrain than on flat roads.
(3) In the local path planning, the authors introduced potential collision area prediction, a temporary target point selection strategy, and the velocity mapping of dynamic obstacles to the improved DWA algorithm. Compared with traditional DWA, the improved DWA algorithm has higher planning efficiency and velocity stability.
(4) CUDA was applied to solve the optimal velocity. In edge computing devices, the solution velocity is increased by 600 times compared to the traditional CPU solution, meeting the requirements for real-time deployment.

The limitations of this work include the following: (1) the environment map is created by the binocular camera with a hole problem, which makes the algorithm treat the hole as a passable area; (2) due to the low tracking accuracy and robustness of dynamic obstacles, the potential collision area prediction accuracy is low, while the algorithm to determine the location and velocity of dynamic obstacles is also affected; (3) the selection of optimal velocity depends on the hardware solution, which increases the hardware requirements. In the next step, multi-beam LIDAR will be used to construct static environment maps. In addition, pedestrian trajectory intention analysis and velocity constraints will be added to dynamic obstacle tracking to reduce the computational scale of velocity planning solutions.

## Figures and Tables

**Table 1:** The range of parameters to be optimized for the PSO-based 3D APF.

Symbol	Range	Symbol	Range
$K_{\mathrm{att}}^{\mathrm{tar}}\in {\mathbb{R}}^{3}$	$\left[\begin{array}{cc} \begin{array}{c} 1.0\\ 1.0\\ 1.0 \end{array} & \begin{array}{c} 100.0\\ 100.0\\ 10.0 \end{array} \end{array}\right]$	$K_{\mathrm{rep}}^{\mathrm{ter}}\in {\mathbb{R}}^{3}$	$\left[\begin{array}{cc} \begin{array}{c} 1.0\\ 1.0\\ 1.0 \end{array} & \begin{array}{c} 100.0\\ 100.0\\ 10.0 \end{array} \end{array}\right]$
$K_{\mathrm{rep}}^{\mathrm{obs}}\in {\mathbb{R}}^{3}$	$\left[\begin{array}{cc} \begin{array}{c} 1.0\\ 1.0\\ 1.0 \end{array} & \begin{array}{c} 100.0\\ 100.0\\ 10.0 \end{array} \end{array}\right]$	*n*	$\left[1.0,2\right]$
$K_{\mathrm{att}}^{\mathrm{ter}}\in {\mathbb{R}}^{3}$	$\left[\begin{array}{cc} \begin{array}{c} 1.0\\ 1.0\\ 1.0 \end{array} & \begin{array}{c} 100.0\\ 100.0\\ 10.0 \end{array} \end{array}\right]$	*step*	$\left[0.1,0.5\right]$

**Table 2:** The fixed parameters of the PSO algorithm and global path planning.

Symbol	Value	Symbol	Value
*c*_0_	0.6	$H_{\mathrm{obs}}^{\max}$	1.2 m
*c*_1_	1.49455	$H_{\mathrm{obs}}^{\min}$	−0.12 m
*c*_2_	1.49455	$H_{\mathrm{ter}}^{\min}$	−0.5 m
$l_{\mathrm{ter}}$	0.3 m	$H_{\mathrm{rep}}^{\min}$	−0.25 m
$l_{\mathrm{ne}}$	1.5 m	$H_{\mathrm{att}}^{\max}$	−0.35 m

**Table 3:** Quantitative comparison of the PSO-based 3D APF’s performance under different parameters settings.

Target	[*α*_1_, *α*_2_, *α*_3_]	Path Smoothness		Terrain Complexity		Number of Iterations
		Mean	Max	Mean	Max	
$T_{1}$	[1, 0, 0]	0.08286	0.31429	0.01125	0.02898	16
	[1, 5, 0]	0.02225	0.08126	0.00915	0.02359	16
	[1, 0, 50]	0.04948	0.29854	0.00612	0.01385	16
	[1, 5, 50]	0.01696	0.06698	0.00627	0.01456	20
*T*_2_	[1, 0, 0]	0.08444	0.48541	0.0096	0.02268	17
	[1, 5, 0]	0.00894	0.09857	0.01114	0.02531	19
	[1, 0, 50]	0.05536	0.41764	0.00562	0.01366	21
	[1, 5, 50]	0.00985	0.11690	0.00639	0.01646	21

**Table 4:** The effective step ratio.

Target		Step	iter	σ
$T_{1}$	Fixed step (reference [29])	0.1	103	1.41
		0.2	43	2.31
		0.3	32	1.67
		0.4	27	1.25
		0.5	30	0.67
	Dynamic step (this article)	Varies in [0.1~0.5]	20	9
*T*_2_	Fixed step (reference [29])	0.1	121	1.36
		0.2	51	2.19
		0.3	37	1.64
		0.4	32	1.21
		0.5	47	0.43
	Dynamic step (this article)	Varies in [0.1~0.5]	21	3.2

**Table 5:** The motion parameters of the quadruped robot.

Symbol	Representation	Value
s	Minimum linear velocity (X-Y-Z)	$\left[-0.15,-0.15,-0.1\right]$ m/s
$v_{max}\in {\mathbb{R}}^{3\times 1}$	Maximum linear velocity (X-Y-Z)	$\left[0.3,0.3,0.15\right]$ m/s
$a_{\max^{v}}$	Maximum linear acceleration (X-Y-Z)	0.3 m/s^2^
$\omega_{\min^{z}}$	Minimum angular velocity (Z)	−0.5235 rad/s
$\omega_{\max^{z}}$	Maximum angular velocity (Z)	0.5235 rad/s
$a_{\max^{\omega }}$	Maximum angular acceleration (Z)	0.5235 rad/s^2^

**Table 6:** The comparative results of the improved DWA and traditional DWA algorithms.

	Mean of Velocity Variance			Path Length (m)
	X-Axis	Y-Axis	Yaw	
traditional-obs1	$1.71\times 10^{-5}$	$5.15\times 10^{-6}$	$3.50\times 10^{-5}$	2.514
improved-obs1	$4.66\times 10^{-6}$	$8.22\times 10^{-7}$	$1.28\times 10^{-5}$	2.021
traditional-obs2	$5.95\times 10^{-6}$	$1.67\times 10^{-5}$	$1.21\times 10^{-5}$	2.327
improved-obs2	$1.51\times 10^{-6}$	$2.92\times 10^{-6}$	$3.69\times 10^{-6}$	1.834

## Data Availability

The raw data supporting the conclusions of this article will be made available by the authors on request.
